# The Innate Immune Response Elicited by Group A Streptococcus Is Highly Variable among Clinical Isolates and Correlates with the *emm* Type

**DOI:** 10.1371/journal.pone.0101464

**Published:** 2014-07-03

**Authors:** Márcia Dinis, Céline Plainvert, Pavel Kovarik, Magalie Longo, Agnès Fouet, Claire Poyart

**Affiliations:** 1 INSERM U 1016, Institut Cochin, Unité FRM “Barrières et Pathogènes”, Paris, France; 2 CNRS UMR 8104, Paris, France; 3 Université Paris Descartes, Sorbonne Paris Cité, Paris, France; 4 Centre National de Référence des Streptocoques, Paris, France; 5 Hôpitaux Universitaires Paris Centre, Site Cochin, Assistance Publique Hôpitaux de Paris, Paris, France; 6 Institut Pasteur, Unité de Biologie des Bactéries Pathogènes à Gram Positif, Paris, France; 7 CNRS 2172, Paris, France; 8 Max F. Perutz Laboratories, University of Vienna, Vienna, Austria; University of South Dakota, United States of America

## Abstract

Group A Streptococcus (GAS) infections remain a significant health care problem due to high morbidity and mortality associated with GAS diseases, along with their increasing worldwide prevalence. Macrophages play a key role in the control and clearance of GAS infections. Moreover, pro-inflammatory cytokines production and GAS persistence and invasion are related. In this study we investigated the correlation between the GAS clinical isolates genotypes, their known clinical history, and their ability to modulate innate immune response. We constituted a collection of 40 independent GAS isolates representative of the *emm* types currently prevalent in France and responsible for invasive (57.5%) and non-invasive (42.5%) clinical manifestations. We tested phagocytosis and survival in mouse bone marrow-derived macrophages and quantified the pro-inflammatory mediators (IL-6, TNF-α) and type I interferon (INF-β) production. Invasive *emm89* isolates were more phagocytosed than their non-invasive counterparts, and *emm89* isolates more than the other isolates. Regarding the survival, differences were observed depending on the isolate *emm* type, but not between invasive and non-invasive isolates within the same *emm* type. The level of inflammatory mediators produced was also *emm* type-dependent and mostly invasiveness status independent. Isolates of the *emm1* type were able to induce the highest levels of both pro-inflammatory cytokines, whereas *emm89* isolates induced the earliest production of IFN-β. Finally, even within *emm* types, there was a variability of the innate immune responses induced, but survival and inflammatory mediator production were not linked.

## Introduction

Group A Streptococcus (GAS, *Streptococcus pyogenes*) is among the most ubiquitous and versatile human bacterial pathogen with major healthcare and economic impacts [1], [2]. This Gram-positive bacterium can cause a broad range of diseases, from self-limiting suppurative infection of the upper respiratory tract (pharyngitis) and skin (impetigo) to deeper and life threatening invasive infections such as toxic shock-like syndrome (STSS), necrotizing fasciitis (NF), with an estimated 500,000 deaths yearly [1], [2].

Since the late 1980's a marked increase of GAS invasive infections has been reported world-wide [1]. Traditionally, GAS have been classified into serological types using M protein serotyping and T protein agglutination assays. Currently, the most widely used typing-method for GAS strains relies on the 5′end of the *emm* gene sequence that encodes the hypervariable amino-terminus region of the M protein [3]. To date more than 200 different *emm* gene types have been defined [4] and the most prevalant *emm* types associated with invasive infections in Europe are *emm1*, *emm28* and *emm89*, with variable distribution worldwide [5], [6]. While correlation between *emm* types and tissue tropism has been reported no link with disease severity has been highlighted except for *emm1* and *emm3* strains, that are associated with NF and STSS [6], [7].

GAS has been described as an extracellular bacterium that circumvent the host immune defenses to survive and persist. It has indeed evolved a broad array of virulence factors to outwit the activities of phagocytic cells [8], [9] and it has developed a number of strategies to avoid or induce an overeaction of the host immune system. Surface components of GAS including a family of M-proteins, the hyaluronic acid capsule, fibronectin and collagen-binding proteins allow the microorganism to adhere, colonise and invade human skin and mucosal tissues under different environmental conditions [2]. The M protein, a fimbrial surface protein, is highly variable and grouped in three classes A-C, D, E [10]. It has an anti-phagocytic activity and it binds to diverse host molecules depending on the class it belongs to among which complement proteins that prevent the alternative complement pathway activation. The bacterium thus evades killing by the polymorphonuclear leucocytes [8], [11]. The hyaluronic acid capsule confers invasiveness *in vivo* through the resistance to phagocytosis by interfering with binding of antibodies [12]. GAS also secretes virulence factors. The SpeB cysteine protease is a crucial virulence factor, wich is able to modulate GAS surface proteins function in colonization and significantly contibutes to tissue destruction in necroziting fasciitis. SpeB can cleave host extracellular matrix proteins, as well as immune system components, and activate matrix metalloproteinases to promote further tissue damage and the release of proapoptotic factors [13]. SLO is a human-specific cytolysin with a range of properties, including the ability to form pores and to prevent the internalization of GAS by lysosomes, thus enhancing the intracellular survival of GAS within epithelial cells [14]. The streptococcal pyrogenic exotoxins (SpeA, SpeC, SpeG to SpeM), streptococcal superantigen A (SSA), and streptococcal mitogenic exotoxin Z (SmeZ) have been identified as superantigens; they are released as toxins that can activate a large proportion of T-cell population, eliciting inflammatory response [2], [15]. The excessive uncoordinate release of cytokines such as IL-1, IL-2, IL-6, TNF-α, IFN, overrides the body, resulting in rash, fever, organ failure, coma and death. Epidemiological studies have tentatively searched for links between *emm* types, superantigen profiles and strain invasiveness but they report different conclusions [16], [17].

The primary line of innate immune defense against most bacterial pathogens consists of resident macrophages and polymorphonuclear neutrophils (PMN's). It has been demonstrated that macrophages have a profound influence in the early host immune defense against GAS [18], [19]. Yet, their role in the early steps of GAS infection remains unclear as they can kill GAS or, in opposite, promote their intracellular survival and even growth.

The high variability of the clinical manifestations is due on the one hand to the diversity of the GAS strains and the other hand to the influence of the host immunogenetic background [2], [20], [21]. Nevertheless, there are no studies addressing whether, and if so how, GAS diversity contributes to differential or even opposite response of the innate immune system. In the present study, we searched whether GAS clinical isolates of distinct genotypes have a differential ability to modulate bone marrow-derived macrophages (BMDMs) response and whether this property correlates with the GAS repertoire of pathologies. For this purpose, we selected a collection of independent invasive and non-invasive clinical isolates representative of the most prevalent *emm* types circulating in France and analyzed *in vitro* how they interact with BMDMs. Criteria of study include bacteria phagocytosis, bacterial survival and the cell cytokine response profile with a specific screen for pro-inflammatory (IL-6 and TNF-α) and immunomodulatory (IFN-β) cytokines.

Our findings demonstrate that innate immune response elicited by Group A Streptococcus is highly variable among clinical isolates and correlates with the *emm* type.

## Material and Methods

### Characterization of GAS isolates

GAS clinical isolates were collected by the CNR-Strep (Table S1) (https://cnr-strep.fr/). The invasive status was defined as the isolation of bacteria from a usually sterile site (e.g. blood, cerebrospinal fluid, bone or joint fluid), or from samples obtained from a non-sterile site in combination with clinical signs of necrotizing fasciitis (NF) or streptococcal toxic shock syndrome (STSS). Bacteraemia was considered to be without focus when no focal symptoms could be identified. Colonization isolates were obtained from pharyngeal carrier obtained from random cases with no clinical symptoms associated. *emm* sequence type was determined by sequencing the variable 5′-end of the *emm* gene and comparing sequences with database of the Center for Disease Control and Prevention [3] (http://www.cdc.gov/ncdidod/biotech/strep/doc.htm). PCR reactions were performed to detect the presence of toxin or superantigen genes or alleles, *speA 1-5*, *speB*, *speC, speJ* and *ssa* as described [6], [22]. The *emm1* invasive strain ATCC 700294 was used as a control [23].

### Bacterial growth conditions

GAS isolates were grown at 37°C without agitation in Todd-Hewitt broth (THB) or in DMEM medium at 37°C under 5% CO_2_ atmosphere. Bacteria were collected in mid-log phase, washed twice with sterile phosphate-buffered saline (PBS), and diluted to the required inoculum and the number of viable bacteria was determined by counting the colony forming units (CFUs) after plating dilutions on TH agar (THA).

### Macrophage cultures and infection assays

Primary bone marrow-derived macrophages (BMDMs) from 6-10 weeks-old female C57Bl/6 mice (Charles River Laboratories), were cultivated in DMEM supplemented with 10% fetal calf serum in the presence of GM-CSF (10 ng/mL) and antibiotics, 30 U/mL penicillin and 30 µg/mL streptomycin as previously described [23]. After 10 days, twenty-four well plates were seeded with 5×10^5^ BMDM's per well and 24 hours later, mid-logarithmic phase bacterial cultures were added at a multiplicity of infection (MOI) of 100 [23]. After 30 min of incubation at 37°C and 5% CO_2_, the non-adherent extracellular bacteria were eliminated removing the culture medium and three washing with sterile PBS. The adherent extracellular bacteria were subsequently killed by incubation, with fresh medium containing 30 U/mL penicillin/and 30 µg/mL streptomycin. At time 0 (T0 which corresponded to 30 min after addition of antibiotics) and at specific time points after, supernatants were collected, centrifuged at 10,000 rpm at 4°C and frozen at −20°C for cytokine quantification and macrophages were lysed with 1 mL sterile distilled water. Serial dilutions of cellular lysates were plated on THA plates and the number of CFUs was determined after 24–48 hours growth at 37°C. For all experiments, 3 independent assays in triplicate were carried out for each bacterial isolate.

### Neutral red uptake assay

The neutral red (NR) uptake assay provides a quantitative estimation of the number of viable cells in a culture [24]. Briefly, after infection cells were washed with warm PBS and 500 µL of NR-medium solution (40 µg/mL in DMEM medium) was added and the cells were further incubated at 37°C, 5% CO_2_ for 2 hours. The NR-medium solution was then removed; cells were washed with PBS and 250 µl neutral red de-staining solution (acetic acid 1%/ethanol 50%) was added. The plates were rapidly shaken until the neutral red had been extracted from the cells and had formed a homogeneous solution. The OD of neutral red extract was measured at 540 nm in a microtiter plate reader spectrophotometer.

### Cytokine quantification

The levels of IL-6, TNF-α and IFN-β were determined, by ELISA, in the supernatants of GAS infected, LPS-stimulated (10 µg/mL, positive control) and unstimulated (negative control) BMDMs. IL-6 and TNF-α were assayed using DuoSet ELISA kits (R&D Systems, Minneapolis, MN). The amount of IFN-β was measured using VeriKine Mouse Interferon Beta Kit (PBL Biomedical laboratories), according to manufacturers' instructions. For all experiments, three independent assays in triplicate were carried out for each bacterial isolate.

### Statistical analysis

Data were analyzed using GraphPad Prism 5.0 (GraphPad Software, San Diego, California). The significance of differences between the values was determined by Mann-Withney test. Significance levels were set at *p≤0.05; **p≤0.01; ***p≤0.005.

### Ethics statement

All of the animal experiments described in this study were conducted in accordance with guidelines of Cochin Institute, in compliance with the European animal welfare regulation (http://ec.europa.eu/environment/chemicals/lab_animals/home_en.html) and were approved by the Institut Cochin animal care and use committee.

## Results

### Selection and genotypic characterization of a relevant collection of GAS clinical isolates

To maximize the relevance of isolate sampling, we selected from the CNR-Strep collection 40 non-redundant GAS isolated from i) different geographical areas, ii) at different time periods, iii) humans of different ages, and iv) responsible for invasive with or without STSS (Inv; n = 23) and non-invasive (pharyngitis, cutaneous infections) infections (NInv; n = 17) (Table S1). These GAS isolates belonged to the *emm1* (n = 15), *emm28* (n = 13) and *emm89* (n = 12) that are the most prevalent *emm* types circulating in France, but also in other European countries and Northern America (Table S1).

We determined the toxin gene profile of these isolates by PCR (Table S1) and found it to be comparable to those described in epidemiological studies [6], [17], [25]. As expected, the *speB* gene was detected in all GAS isolates whatever the *emm* type. All *emm1* isolates contained *speA* and *speJ*. *speA1–3* alleles were found in all *emm1* isolates, with the exception of the M1 NInv1 isolate which harbors the *speA5* allele. Among the 40 isolates, those from the *emm1* group are the only ones to display such uniformity for these two toxin genes and which is invasiveness status independent. Furthermore, isolates M1 Inv7, M1 Inv8 and M1 NInv5 also harbor *speC*. All *emm28* isolates carry the *speC* gene and half of them, equally distributed among the invasive and non-invasive isolates, also harbor *speJ*. The *emm89* isolates had, with the exception of M89 Inv2, M89 Inv5 and M89 NInv2, *speC*, equally distributed among invasive and non-invasive isolates. These results confirmed that the toxin gene profile is quite similar within each *emm* type, but differs between *emm* types. Interestingly, the toxin gene profile of the isolates was independent of the clinical manifestation.

### Macrophage GAS phagocytosis depends both upon *emm* type and the invasiveness status of the isolate, whereas only *emm* type correlates with survival

**Figure 1:**
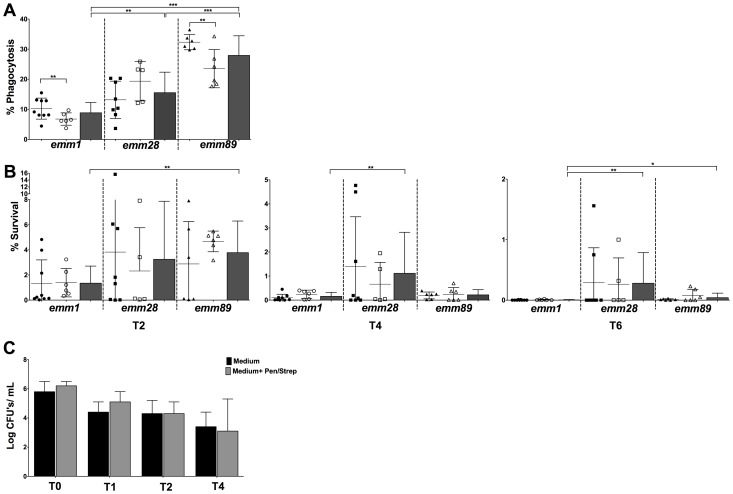
Phagocytosis and survival of GAS clinical isolates in BMDMs. Cells were infected with GAS as described in Material and Methods. (**A**) Percentage of phagocytosis of all (black bars), invasive (black symbols) and non-invasive (white symbols) GAS strains of different *emm* types. The results are expressed as the percentage of bacterial CFUs recovered after 30 min post-antibiotics treatment relative to the initial inoculum. (**B**) Bacterial survival experiments were carried out as described in the Material and Methods and expressed as the percentage of phagocytosed bacteria that survived. (**C**) Intracellular bacteria were not killed by extracellular antibiotics. Cells were infected with GAS as described and after washing, either medium with antibiotics (ATB) (black) or medium alone (dash) was added to cells. The results represent the mean ± SD of three independent experiments, with significance levels indicated between given isolates from the same *emm* type or in between *emm* types (*p≤0.05; **p≤0.01; ***p≤0.005).

Macrophages are critical host defense cells involved directly in bacterial clearance and also in alerting other immune system components to invading pathogens. We questioned if all GAS isolates showed the same behavior in terms of macrophage's uptake and bacterial survival. To this end, BMDMs were incubated with each one of the 40 GAS isolates and the phagocytosis rate of each isolate was compared by determining the percentage of bacterial CFUs recovered after 30 min post-antibiotics treatment relative to the initial inoculum (Fig. 1A, Table S2). We observed that the percentage of GAS phagocytosis varied significantly depending on the *emm* type and within some *emm* types according to the invasiveness status. Indeed, invasive (Inv, black symbol) *emm1* and *emm89* isolates were significantly more phagocytosed than non-invasive (NInv, white symbol) isolates of the same *emm* type (Fig.1A). Also, *emm89* invasive isolates were significantly more heavily phagocytosed than the other invasive isolates (*emm1* p<0.0022, *emm28* p = 0.0007). In addition, while the range in the phagocytosis rate by BMDM cells varied substantially between *emm28* invasive isolates it was limited for *emm89* and *emm1* invasive isolates. Interestingly while invasive isolates were found more prone to phagocytosis, the *emm1* strains that are considered among the most virulent because often associated with STSS or NF were, in contrast, the less phagocytosed.

We then tested whether survival of GAS clinical isolates in BMDMs within a 6-hour time window varies with the *emm* type or with the isolate-associated clinical manifestations (Fig. 1B, Table S2). Survival of any given isolate decreased over time (Table S2). In contrast to the phagocytosis results, no significant differences were seen, within each *emm*-type, between invasive (black symbols) and non-invasive (white symbols) isolates (Fig. 1B). Interestingly, the *emm89* isolates survived better than their *emm1* counterparts at T2 and T6. Noteworthy, although the percentages of survival of the *emm89* isolates are only slightly higher, the difference in phagocytosis leads to far more *emm89* bacteria surviving at all time points (Table S2). The *emm28* isolates, be they invasive or non-invasive, showed the highest dispersion in terms of survival. Nevertheless, *emm28* isolates survived better than *emm1* isolates at T4 and T6. One *emm28* strain, M28 Inv6 appeared to be less phagocytosed and to be cleared faster than all other strains. However, the inoculum used for this strain was one log_10_ lower than with all other strains. The smaller MOI may account for this difference.

Because added streptomycin can in some instance kill intra-cellular bacteria, we checked that in the conditions used, this was not the case. The experiment was carried out with the control *emm1* invasive ATCC 700294 strain using the same protocol except that after the washing step in presence of antibiotics (T0), either medium alone or medium supplemented with antibiotics was added and survival was followed by determination of the CFU counts at T0, 1 h (T1), 2 h (T2) and 4 h (T4) p.i. (Fig. 1C). The number of the ATCC 700294 CFUs was similar at each time point, regardless of the presence of antibiotics, indicating that extracellular antibiotics did not kill the intracellular bacteria.

Collectively these results indicate that whereas bacterial uptake depends on *emm* type and somewhat on the isolate invasiveness status, intracellular bacteria survival does not depend on the invasiveness status but rather on the *emm* type of the isolate.

### The immune mediator secretion is also correlated with GAS *emm* type

Since GAS induces secretion of pro-inflammatory cytokines during the innate immune response [26], we tested whether the profile of secreted inflammatory mediators correlated with the GAS *emm* type or with the isolate-associated clinical manifestation. We first tested whether infected BMDMs were damaged by intracellular bacteria by measuring the ability of BMDMS infected with the control *emm1* invasive ATCC 700294 strain as well as with three clinical isolates from our collection, M1 Ninv1, M28 Inv5 and M89 NInv2, to retain the supravital dye neutral red (Fig. S1). *emm1* strains have been described to induce macrophage apoptosis [26]. The amount of intracellular neutral red was similar in non-infected BMDMs and in all infected BMDMs, indicating that the macrophages were not damaged during the course of this experiment.

**Figure 2:**
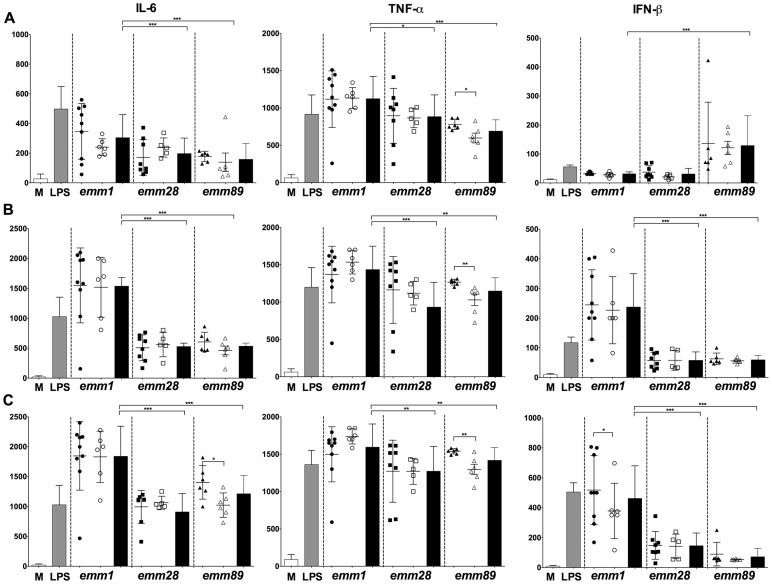
GAS clinical isolates induced pro-inflammatory mediator and IFN-β secretion by infected BMDMs. Graphics represent IL-6, TNF-α and IFN-β quantification in the cell culture supernatant at T2h (**A**), T4h (**B**) and T6h (**C**) after infection by the different *emm* type isolates. The mean values immune mediator productions induced by all (black bars), invasive (black symbols) and non-invasive (white symbols) of isolates of each *emm* type are shown. To note, the scales are different, within the IL-6 data in panel A and, within the IFN-β data in panel C. The results represent the mean ± SD of 3 independent experiments; with significance levels indicated between *emm1* and other *emm* types by stars above the corresponding black bar and within *emm* types by stars above a line overlapping the corresponding the black and white symbols (*p≤0.05; **p≤0.01; ***p≤0.005).

To test a putative correlation between the profile of secreted inflammatory mediators and the bacterial characteristics, culture supernatants of BMDMs loaded or not with GAS and of BMDMs stimulated with LPS (10 µg/mL) were collected at different time points and cytokine levels were measured using ELISA (Fig. 2). All groups of isolates were able to induce the production of early mediators IL-6 and TNF-α. Results revealed that the pro-inflammatory mediator production levels were all dependent on the isolate *emm* type.

First, the *emm1* type isolates induced higher levels of IL-6 secretion by BMDMs than isolates from any other *emm* type throughout the experiment. IL-6 production by GAS-loaded cells was similar for both invasive and non-invasive isolates, except for the *emm89* isolates at T6. IL-6 levels increased approximately three-fold and two- to three-fold in all infected BMDMs between T2 and T4 p.i., and T4 and T6 p.i., respectively.

Secondly, the *emm1* type isolates induced higher levels of TNF-α secretion by BMDMs than isolates from any other *emm* type at all time points. The secreted TNF-α levels slightly increased over time p.i. for all isolate groups. TNF-α secretion was not significantly different in cells loaded with invasive or non-invasive isolate except for the *emm89* infected BMDMs for which invasive isolates triggered higher levels of TNF-α secretion as compared to non-invasive *emm89* isolates at all time points.

Analysis at the individual isolate level indicated that *emm89* GAS throughout the experiment yielded the less scattered values of all (Fig. 2, Table S3). Isolates that elicited high-production, compared to the mean value within their *emm* type, of one cytokine did not necessarily elicit a high production of the other: there was no link between the relative levels of IL-6 and TNF-α induced productions. No correlation existed between CFU counts and the induced production level of IL-6 and TNF-α, with one exception, M28 Inv6 (Table S2, Table S3). This isolate, which was the only *emm28* isolate to be cleared at T2, induced one of the lowest IL-6 and TNF-α productions. Finally, one isolate, M1 NInv4, which was not cleared, elicited a lower production, than all other *emm1* isolates, of both IL-6 and TNF-α throughout the course of this experiment.

The ability of GAS *emm1* to induce IFN-β production and its role in host protection have been recently reported [23], [27]. We thus investigated IFN-β production by BMDMs infected by the clinical isolates from our collection (Fig. 2). As with the pro-inflammatory cytokine production, the mean values of IFN-β secretion elicited by invasive and non-invasive isolates within each *emm* type were similar, with the exception at T6 of the *emm1*-infected BMDMs, where the invasive isolates elicited the highest production. The IFN-β production kinetics differed depending on the *emm* type. The *emm89* isolate-infected BMDMs had an early production of IFN-β, which was the highest of all infected BMDMs at T2 and which then decreased, whereas for all other isolates the elicited production was barely detectable before T4 or T6. From T4 onwards, the *emm1* type isolates induced the highest production.

Analysis at the individual isolate level (Fig. 2, Table S3) indicated that the amount of IFN-β induced by each isolate, except the *emm89* isolates, increased with time. In contrast the level of secreted IFN-β decreased with all but one of the *emm89* isolates. Again, the M28 Inv6 isolate elicited the lowest IFN-β production throughout time.

The levels of pro-inflammatory cytokines and type I interferon produced is clearly dependent on the isolate *emm* type but seldom dependent on the invasive status of the isolates.

## Discussion

The interaction between GAS and the host innate immune response has been studied *in vitro* and *in vivo* [8], [9], [18], [19], [28], [29],[30],[31],[32] but most of the time, these studies were performed with one strain from a given *emm* type leading to conclusions that were not necessarily representative and relevant of a given population. Genomic analyses of multiple GAS strains have been conducted to search for gene linkage and tissue tropism or disease severity, however *in vitro* experiments supporting the results have not yet been reported [7], [16], [17]. Our aim was thus to compare *in vitro* key features of the early innate immune response elicited by a relevant collection of GAS isolates (n = 40) from different *emm* types and invasiveness status.

We first assessed whether there was a correlation between the genotypic toxin profile and the invasiveness status of the isolates. The toxin profile showed high conservation within each *emm* type and variation between *emm* types and no correlation was found with the isolate invasiveness status. Similar results were obtained while studying the presence of 9 and 11 superantigens and 11 superantigens and different alleles of *speA*, in 87, 107 and 291 isolates, respectively [16], [17], [25]. However, in that carried out on 291 clinical isolates (194 colonization and 97 invasive isolates), the *speA1-speA3* alleles, as well as the *speJ* gene were found more frequently in invasive than colonization isolates [16]. Our study shows that neither *speA* allele nor the presence of *speJ* could be linked to the clinical manifestation. It is therefore likely that the differences observed might be due to the expression level of superantigens which can be modulated by bacterial or human host determinants.

The role of macrophages in GAS diseases may be dual as they can kill the bacteria but they can also be used as a Trojan horse in which bacteria survive [19], [33]. The first step benefits the host while in contrast the second benefits the bacterium. Herein we report that invasive isolates from *emm* type *emm89*, were more phagocytosed than their non-invasive counterparts. *emm1* invasive isolates have been shown to persist and even multiply in the macrophages. When coupled to a more efficient phagocytosis, these features might contribute to the increased invasion capacity of some *emm1* isolates. The invasive *emm89* isolates which were the most successfully phagocytosed isolates in this study also survived intracellularly slightly better than others, suggesting that they might hijack the macrophages to their own benefit, in particular for persistence. It would be interesting to study whether *emm89* isolates elicit particularly persistent or recurring GAS infections while our observation suggests that they could be responsible for antibiotic treatment failure observed in some GAS infections. Finally, *emm28* isolates, that are associated with puerperal fever, are also able to survive more than *emm1* isolates in the macrophage [6]. Survival or death of intracellular bacteria may be a consequence of the entry pathway [19]. The entry pathway, permitting GAS survival, and bacterial factors promoting it are currently unknown. Among bacterial factors involved in survival are the M1 protein shown to impair proper fusion of the phagosome with lysosomes and SLO, together with NAD-glyohydrolase, reported to protect GAS from xenophagic killing [14]. Since the *emm89* isolates, on the whole, survived better than isolates from the *emm1 emm* type, it would be interesting to test whether the M89 protein or the associated M-like proteins interfere with the host phagosomal-lysosomal pathway. Interestingly, the *emm89* isolates induced a more rapid secretion of IFN-β as well as a weaker IL-6 and TNF-α by macrophages than isolates from the other *emm* types. One hypothesis to account for this difference is that the M89 protein or the M-like proteins could, like M1 but more efficiently, induce a suppression of inflammatory signals. Alternatively and not exclusively, the early IFN-β secretion may control the pro-inflammatory response, limiting it. The reason for the decrease in IFN-β production by the *emm89*-infected BMDMs as soon as T4 is currently unknown.

In our experiments, the survival property of the isolates was not linked to the invasive status of the isolates. However, *in vivo*, the recruitment of other phagocytic cells may interfere with the tissue invasion properties of the isolate. Since qualitative and quantitative differences in the cytokine secreted between isolates have been detected in our *in vitro* assays, these are likely to modulate phagocytic cell as well as non-phagocytic cell recruitment and functions and therefore to contribute to the invasion phenotype. Finally, studies have demonstrated that the same streptococcal strain could cause infections with diverse severity in different individuals, emphasizing the influence of the host genetic factors, most likely involved in the defense responses, on the outcome of infections [34].

In our study we observed that GAS had different pathogenesis mechanisms depending on the *emm* type. The *emm1* isolates were able to induce an early overwhelming pro-inflammatory cytokines production. *In vivo*, this may drive the host system in an inflammatory loop that would be damaging for the host.

In the case of *emm89* isolates, that were phagocytized and survived longer than any other *emm* type isolates, the mechanism might involve pro-inflammatory signals but at later time points, and the initial interferon type I production may interfere with the recruitment of other innate immune cells including neutrophils, as already reported [27]. Of note, the isolates that have longer-term intracellular survival potential have higher chances to avoid the antibiotics therapeutics that are commonly prescribed for the treatment of GAS infections.

With the goal of assaying the existence of differences depending on the invasiveness status or on the genotype of the strains, the experiments were carried out with murine BMDMs. Although using mouse macrophages for studying this human pathogen has some limitations, this model has previously been successfully used and has the advantage that the cells are not donor dependent [16]. Furthermore, the mouse proinflammatory response mimics that observed in patients with severe invasive infections. Plasma proteins such as fibrinogen could also be added to better mimic the *in vivo* situation. Our study has enabled us, in addition to draw conclusion as to the importance of the *emm* type on all innate immunity events tested, to point out to strains that are representative of all strains from their groups and others which are atypical. These strains, in limited number, may now be employed to further study the mechanisms involved in the host-pathogen interaction using human cells.

In conclusion, for all aspects of the innate immune response analyzed, the *emm* type has more influence than the invasiveness status of the isolate.

## Supporting Information

Figure S1 **BMDMS viability is not altered during the experiments.** BMDMS viability is not altered during the experiments. The BMDMs were infected using MOI = 100 for 30 min at 37°C; afterwards cells were washed and incubated with medium plus antibiotics. At each time point the neutral red medium was added, and after 2 h incubation at 37°C the plates were washed and the dye was extracted with acidified ethanol solution. A decrease in color was quantified at 540 nm. The percentage of viable cells was calculated as follows, the mean value from wells without cells was subtracted from the other wells, and the values of treated cultures were referred to control uninfected cultures. Values represent the mean ± SD of percentage of neutral red uptake at different time points of two wells per treatment and correspond to one representative experiment of three independent experiments.
(TIF)

Table S1 **Characteristics of GAS isolates used in this study.**
(DOC)

Table S2 **Phagocytosis and intracellular survival.**
(DOC)

Table S3 **Innate immune modulators production in the BMDMs cultures.**
(DOCX)
