# Supplementary figures and images for: The Innate Immune Response Elicited by Group A Streptococcus Is Highly Variable among Clinical Isolates and Correlates with the emm Type
Source: PLoS One. 2014 Jul 3;9(7):e101464. doi: 10.1371/journal.pone.0101464 (PMC4081719; doi:10.1371/journal.pone.0101464)

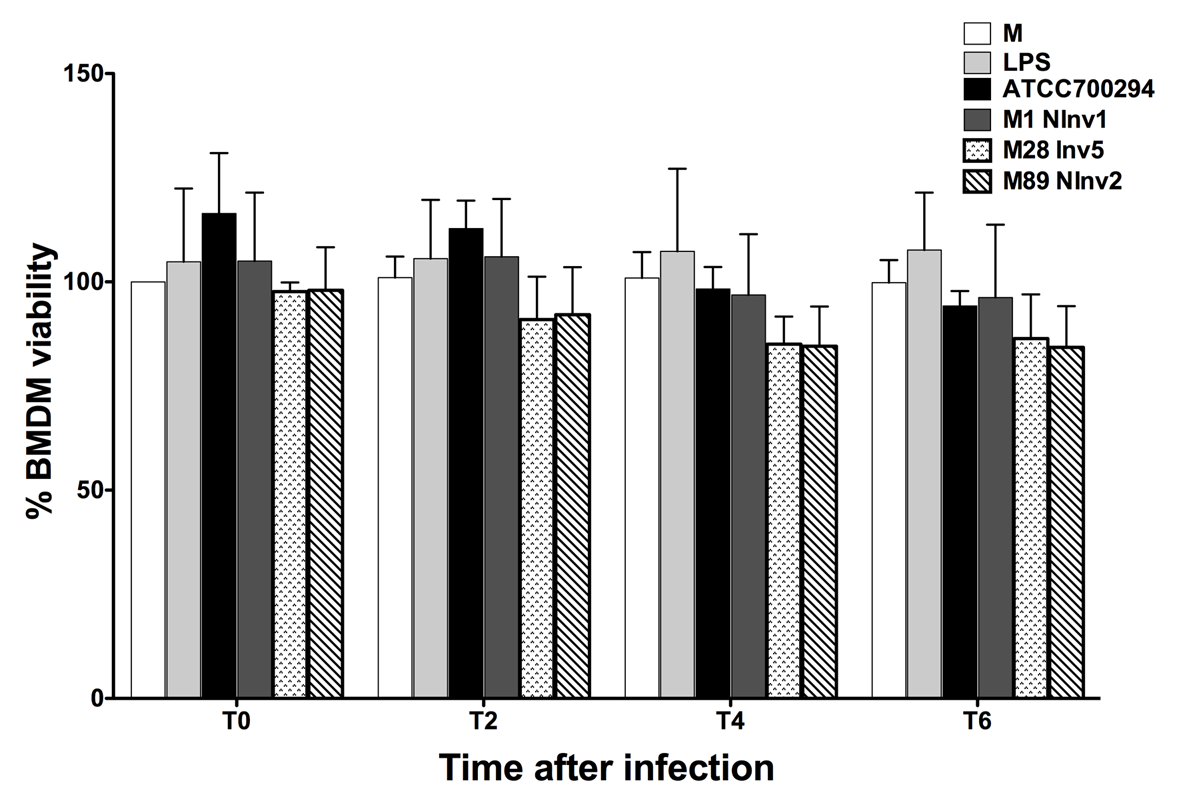

Supplement: Figure S1 — BMDMS viability is not altered during the experiments. BMDMS viability is not altered during the experiments. The BMDMs were infected using MOI = 100 for 30 min at 37°C; afterwards cells were washed and incubated with medium plus antibiotics. At each time point the neutral red medium was added, and after 2 h incubation at 37°C the plates were washed and the dye was extracted with acidified ethanol solution. A decrease in color was quantified at 540 nm. The percentage of viable cells was calculated as follows, the mean value from wells without cells was subtracted from the other wells, and the values of treated cultures were referred to control uninfected cultures. Values represent the mean ± SD of percentage of neutral red uptake at different time points of two wells per treatment and correspond to one representative experiment of three independent experiments. (TIF) [file pone.0101464.s001.tif]
