# Supplementary material for: The Innate Immune Response Elicited by Group A Streptococcus Is Highly Variable among Clinical Isolates and Correlates with the emm Type
Source: PLoS One. 2014 Jul 3;9(7):e101464. doi: 10.1371/journal.pone.0101464 (PMC4081719; doi:10.1371/journal.pone.0101464)
Supplement: Table S1 — Characteristics of GAS isolates used in this study. (DOC) [file pone.0101464.s002.doc]

**Table S1.** Characteristics of GAS isolates used in this study

| **Reference number** | **M type** | **Strain^a^** | **Diagnostic^b^** | **Toxin genes** |
| --- | --- | --- | --- | --- |
| 20030096 | M1 | M1 Inv1 | NF+STSS | *speA1-3, speB, speJ* |
| 20030192 | M1 | M1 Inv2 | NF+STSS | *speA1-3, speB, speJ* |
| 20040420 | M1 | M1 Inv3 | STSS | *speA1-3, speB, speJ* |
| 20040427 | M1 | M1 Inv4 | NF | *speA1-3, speB, speJ* |
| 20040562 | M1 | M1 Inv5 | NF+STSS | *speA1-3, speB, speJ* |
| 20050062 | M1 | M1 Inv6 | NF+STSS | *speA1-3, speB, speJ* |
| 20070592 | M1 | M1 Inv7 | NF+STSS | *speA1-3, speB, speC, speJ* |
| 20070779 | M1 | M1 Inv8 | NF+STSS | *speA1-3, speB, speC, speJ* |
| 20070902 | M1 | M1 Inv9 | Bacteremia +STSS | *speA1-3, speB, speJ* |
| 20040036 | M1 | M1 NInv1 | Pharyngeal carrier | *speA5, speB, speJ* |
| 20050138 | M1 | M1 NInv2 | Pharyngeal carrier | *speA1-3, speB, speJ* |
| 20050374 | M1 | M1 NInv3 | Pharyngeal carrier | *speA1-3, speB, speJ* |
| 20070445 | M1 | M1 NInv4 | Vaginal colonization | *speA1-3, speB, speJ* |
| 20080126 | M1 | M1 NInv5 | Pharyngeal carrier | *speA1-3, speB, speC, speJ* |
| 20060057 | M28 | M28 Inv1 | Arthritis+STSS | *speB, speC* |
| 20060831 | M28 | M28 Inv2 | DHN+STSS | *speB, speC* |
| 20070586 | M28 | M28 Inv3 | Bacteremia | *speB, speC* |
| 20070662 | M28 | M28 Inv4 | Bacteremia | *speB, speC, speJ* |
| 20070748 | M28 | M28 Inv5 | Pleuro-pneumopathology | *speB, speC, speJ* |
| 20070963 | M28 | M28 Inv6 | DHN+STSS | *speB, speC, speJ* |
| 20071009 | M28 | M28 Inv7 | NF+STSS | *speB, speC, speJ* |
| 20080319 | M28 | M28 Inv8 | DHN+STSS | *speB, speC* |
| 20040035 | M28 | M28 NInv1 | Pharyngitis | *speB, speC* |
| 20040037 | M28 | M28 NInv2 | Conjunctivitis | *speB, speC* |
| 20060811 | M28 | M28 NInv3 | Colonization | *speB, speC* |
| 20080184 | M28 | M28 NInv4 | Pharyngeal carrier | *speB, speC, speJ* |
| 20080408 | M28 | M28 NInv5 | Colonization | *speB, speC, speJ* |
| 20030451 | M89 | M89 Inv1 | DHN+STSS | *speB, speC* |
| 20050003 | M89 | M89 Inv2 | DHN+Endocarditis | *speB* |
| 20060051 | M89 | M89 Inv3 | DHN | *speB, speC* |
| 20070057 | M89 | M89 Inv4 | DHN+STSS | *speB, speC* |
| 20070249 | M89 | M89 Inv5 | DHN+STSS | *speB* |
| 20070884 | M89 | M89 Inv6 | DHN+STSS | *speB, speC* |
| 20070937 | M89 | M89 NInv1 | Pharyngeal carrier | *speB, speC* |
| 20080105 | M89 | M89 NInv2 | Superinfection | *speB* |
| 20080199 | M89 | M89 NInv3 | Superinfection | *speB, speC* |
| 20080274 | M89 | M89 NInv4 | Pharyngeal carrier | *speB, speC* |
| 20080311 | M89 | M89 NInv5 | Pharyngeal carrier | *speB, speC* |
| 20080412 | M89 | M89 NInv6 | Pharyngeal carrier | *speB, speC* |

^a^ Invasive (Inv); Non-invasive (NInv)

^b^ Necrotizing fasciitis (NF); Streptococcal Toxic Shock Syndrome (STSS); Dermo-hypodermitis necrotizing (DHN)
