# Supplementary material for: The Innate Immune Response Elicited by Group A Streptococcus Is Highly Variable among Clinical Isolates and Correlates with the emm Type
Source: PLoS One. 2014 Jul 3;9(7):e101464. doi: 10.1371/journal.pone.0101464 (PMC4081719; doi:10.1371/journal.pone.0101464)
Supplement: Table S2 — Phagocytosis and intracellular survival. (DOC) [file pone.0101464.s003.doc]

| **Table S2** – Phagocytosis and intracelluler survival | | | | | |
| --- | --- | --- | --- | --- | --- |
| **Log cfus/ mL** | | | | | |
| **Isolate** | **Inoculum** | **T0** | **T2** | **T4** | **T6** |
| M1 Inv1 | 7.52 ± 0.05 | 6.26 ± 0.06 | 3.30 ± 0.08 | 2.45 ± 0.21 | 0.00 ± 0.00 |
| M1 Inv2 | 7.61 ± 0.20 | 6.49 ± 0.14 | 4.82 ± 0.12 | 3.75 ± 0.39 | 2.57 ± 0.05 |
| M1 Inv3 | 7.34 ± 0.01 | 6.41 ± 0.07 | 3.49 ± 0.08 | 3.43 ± 0.06 | 0.00 ± 0.00 |
| M1 Inv4 | 7.39 ± 0.02 | 6.41 ± 0.21 | 5.01 ± 0.14 | 4.06 ± 0.14 | 2.24 ± 0.13 |
| M1 Inv5 | 7.45 ± 0.05 | 6.21 ±0.04 | 3.34 ± 0.07 | 3.14 ±0.04 | 0.00 ±0.00 |
| M1 Inv6 | 7.47 ± 0.01 | 6.41 ± 0.01 | 2.76 ± 0.01 | 2.08 ± 0.12 | 0.00 ± 0.00 |
| M1 Inv7 | 7.32 ± 0.02 | 5.92 ± 0.02 | 3.53 ± 0.47 | 0.00 ± 0.00 | 0.00 ± 0.00 |
| M1 Inv8 | 7.40 ± 0.10 | 6.21 ± 0.03 | 4.89 ±0.07 | 2.60 ±0.12 | 0.00 ± 0.00 |
| M1 Inv9 | 7.34 ± 0.03 | 6.20 ± 0.09 | 3.56 ± 0.03 | 2.19 ± 0.20 | 0.00 ± 0.00 |
| M1 NInv1 | 7.46 ± 0.11 | 6.28 ± 0.16 | 4.37 ± 0.24 | 3.88 ± 0.22 | 2.60 ± 0.42 |
| M1 NInv2 | 7.50 ± 0.20 | 6.09 ± 0.08 | 4.60 ± 0.17 | 3.63 ± 0.76 | 1.00 ± 0.71 |
| M1 NInv3 | 7.39 ± 0.01 | 6.22 ± 0.01 | 4.57 ± 0.03 | 3.64 ± 0.67 | 0.00 ± 0.00 |
| M1 NInv4 | 7.33 ± 0.03 | 5.87 ± 0.08 | 3.75 ± 0.03 | 3.45 ± 0.21 | 0.00 ± 0.00 |
| M1 NInv5 | 7.30 ± 0.02 | 6.11 ± 0.11 | 3.64 ± 0.46 | 2.73 ± 0.90 | 0.83 ±0.37 |
| M1 NInv6 | 7.40 ± 0.21 | 6.09 ± 0.10 | 3.80 ± 0.59 | 2.47 ± 0.72 | 1.15 ± 0.67 |
|  |  |  |  |  |  |
| M28 Inv1 | 7.33 ± 0.01 | 6.21 ± 0.03 | 4.33 ± 0.05 | 3.49 ± 0.02 | 0.00 ± 0.00 |
| M28 Inv2 | 7.64 ± 0.23 | 6.58 ± 0.22 | 4.84 ± 0.11 | 3.49 ± 0.06 | 0.00 ± 0.00 |
| M28 Inv3 | 7.57 ± 0.30 | 6.60 ± 0.26 | 5.39 ± 0.24 | 4.81 ± 0.27 | 4.48 ± 0.36 |
| M28 Inv4 | 7.00 ± 0.02 | 4.86 ± 0.03 | 4.62 ± 0.01 | 4.51 ± 0.01 | 0.83 ± 0.01 |
| M28 Inv5 | 7.46 ± 0.10 | 6.60 ± 0.10 | 5.80 ± 0.26 | 5.28 ± 0.21 | 4.80 ± 0.53 |
| M28 Inv6 | 6.00 ± 0.02 | 3.29 ± 0.12 | 0.00 ± 0.00 | 0.00 ± 0.00 | 0.00 ± 0.00 |
| M28 Inv7 | 7.61 ± 0.21 | 6.31 ± 0.14 | 2.67 ± 0.33 | 0.00 ± 0.00 | 0.00 ± 0.00 |
| M28 Inv8 | 7.34 ± 0.02 | 6.42 ±0.08 | 3.08 ± 0.09 | 0.00 ± 0.00 | 0.00 ± 0.00 |
| M28 NInv1 | 7.40 ± 0.10 | 6.66 ± 0.07 | 5.56 ±0.14 | 4.95 ± 0.24 | 4.66 ± 0.26 |
| M28 NInv2 | 7.29 ± 0.02 | 6.39 ± 0.14 | 4.93 ± 0.18 | 4.51 ± 0.44 | 3.90 ± 0.61 |
| M28 NInv3 | 7.23 ± 0.01 | 6.38 ± 0.15 | 3.06 ± 0.08 | 0.00 ± 0.00 | 0.00 ± 0.00 |
| M28 NInv4 | 7.48 ± 0.15 | 6.66 ± 0.07 | 3.73 ± 0.06 | 3.24 ± 0.06 | 0.00 ± 0.00 |
| M28 NInv5 | 7.51 ± 0.05 | 6.71 ± 0.24 | 3.67 ± 0.06 | 3.26 ± 0.08 | 2.37 ± 0.11 |
|  |  |  |  |  |  |
| M89 Inv1 | 7.35 ± 0.02 | 6.82 ± 0.05 | 5.72 ± 0.02 | 4.18 ± 0.03 | 3.26 ± 0.06 |
| M89 Inv2 | 7.40 ± 0.10 | 6.78 ± 0.10 | 5.63 ± 0.19 | 4.18 ± 0.63 | 3.11 ± 0.09 |
| M89 Inv3 | 7.45 ± 0.15 | 6.81 ± 0.19 | 5.42 ± 0.29 | 4.20 ± 0.38 | 2.65 ± 0.03 |
| M89 Inv4 | 7.35 ± 0.02 | 6.77 ± 0.03 | 5.09 ± 0.12 | 4.36 ± 0.07 | 2.69 ± 0.20 |
| M89 Inv5 | 7.36 ± 0.03 | 6.79 ± 0.09 | 3.86 ± 0.69 | 3.24 ± 0.97 | 2.24 ± 0.04 |
| M89 Inv6 | 7.30 ± 0.02 | 6.86 ± 0.07 | 5.68 ± 0.14 | 3.21 ± 0.75 | 2.47 ± 0.03 |
| M89 NInv1 | 7.48 ± 0.05 | 6.56 ± 0.03 | 5.11 ± 0.04 | 2.35 ± 0.41 | 0.00 ± 0.00 |
| M89 NInv2 | 7.50 ± 0.07 | 6.54 ± 0.23 | 5.47 ± 0.08 | 4.11 ± 0.16 | 3.81 ± 0.20 |
| M89 NInv3 | 7.46 ± 0.05 | 6.73 ± 0.07 | 5.13 ± 0.42 | 4.57 ± 0.23 | 4.09 ± 0.22 |
| M89 NInv4 | 7.40 ± 0.02 | 6.59 ± 0.04 | 4.77 ± 0.07 | 4.13 ± 0.26 | 3.27 ± 0.07 |
| M89 NInv5 | 7.37 ± 0.04 | 6.68 ± 0.02 | 3.17 ± 0.11 | 2.10 ± 0.01 | 0.00 ± 0.00 |
| M89 NInv6 | 7.45 ± 0.02 | 6.83 ± 0.15 | 4.37 ± 0.23 | 3.00 ± 0.04 | 0.00 ± 0.00 |

The values are the means ± SD of three independent experiments
