# Supplementary material for: The Innate Immune Response Elicited by Group A Streptococcus Is Highly Variable among Clinical Isolates and Correlates with the emm Type
Source: PLoS One. 2014 Jul 3;9(7):e101464. doi: 10.1371/journal.pone.0101464 (PMC4081719; doi:10.1371/journal.pone.0101464)
Supplement: Table S3 — Innate immune modulators production in the BMDMs cultures. (DOCX) [file pone.0101464.s004.docx]

**Table S3 –** Innate immune modulators production in the BMDMs cultures.

| **BMDMS supernatants cytokines (pg/ mL)** | | | | | | | | | |
| --- | --- | --- | --- | --- | --- | --- | --- | --- | --- |
|  | **T2** | | | **T4** | | | **T6** | | |
| **Strain** | **IL-6** | **TNF-α** | **IFN-β** | **IL-6** | **TNF-α** | **IFN-β** | **IL-6** | **TNF-α** | **IFN-β** |
| **M1 Inv1** | 560.0 ± 3.9 | 1510.0 ± 27.4 | 40.0 ± 2.6 | 1978.4 ± 39.4 | 1680.0 ± 53.9 | 340.6 ± 24.9 | 2400.0 ± 22.0 | 1700.0 ± 31.3 | 806.3 ± 3.3 |
| **M1 Inv2** | 516.0 ± 1.6 | 1000.0 ± 13.9 | 30.0 ± 2.1 | 1603.4 ± 100.1 | 1200.0 ± 20.5 | 200.0 ± 31.8 | 1998.6 ± 20.2 | 1300.0 ± 42.2 | 500.0 ± 20.9 |
| **M1 Inv3** | 400.0 ± 1.9 | 1300.0 ± 16.9 | 30.00 ± 1.4 | 2100.0 ± 105.1 | 1600.0 ± 37.8 | 400.0 ± 8.5 | 2200.0 ± 66.6 | 1650.0 ± 29.5 | 800.0 ± 26.9 |
| **M1 Inv4** | 56.6 ± 1.8 | 258.5 ± 6.2 | 35.4 ± 9.3 | 155.4 ± 4.3 | 448.5 ± 13.1 | 57.2 ± 2.5 | 466.5 ± 34.3 | 589.9 ± 16.8 | 168.2 ± 3.8 |
| **M1 Inv5** | 502.8 ± 6.3 | 1445.5 ± 54.4 | 36.7 ± 6.0 | 1969.3 ± 43.0 | 1619.2 ± 17.7 | 404.8 ± 13.7 | 2154.6 ± 79.2 | 1650.0 ± 88.0 | 753.3 ± 3.9 |
| **M1 Inv6** | 458.1 ± 5.9 | 1389.2 ± 27.1 | 40.0 ± 3.0 | 2055.8 ± 43.7 | 1545.4 ± 64.2 | 250.0 ± 15.3 | 2165.0 ± 78.9 | 1612.5 ± 17.7 | 500.0 ± 10.3 |
| **M1 Inv7** | 122.3 ± 10.4 | 976.5 ± 5.34 | 31.9 ± 3.3 | 1031.5 ± 48.8 | 1434.6 ± 54.7 | 220.7 ± 14.4 | 1587.3 ± 62.3 | 1794.6 ± 71.7 | 342.5 ± 4.5 |
| **M1 Inv8** | 161.8 ± 4.8 | 1041.1 ± 5.34 | 30.4 ± 3.1 | 1478.2 ± 28.5 | 1286.2 ± 53.6 | 128.4 ± 5.73 | 1842.8 ± 74.4 | 1499.9 ± 86.0 | 291.6 ± 6.3 |
| **M1 Inv9** | 343.1 ± 15.4 | 1142.1 ± 7.61 | 30.0 ± 2.6 | 1579.8 ± 56.7 | 1506.3 ± 14.5 | 200.0 ± 8.0 | 1800.0 ± 90.5 | 1688.0 ± 68.1 | 500.0 ± 14.5 |
| **M1 NInv1** | 240.9 ± 26.9 | 952.7 ± 23.3 | 15.0 ± 0.7 | 1648.5 ± 18.8 | 1300.0 ± 50.5 | 427.8 ± 16.2 | 2000.0 ± 87.3 | 1700.0 ± 52.6 | 696.9 ± 11.1 |
| **M1 NInv2** | 330.0 ± 3.5 | 1340.0 ± 20.4 | 30.0 ± 5.3 | 1962.1 ± 25.8 | 1700.0 ± 51.0 | 200.0 ± 17.5 | 2276.3 ± 68.8 | 1750.0 ± 50.1 | 375.0 ± 7.3 |
| **M1 NInv3** | 190.0 ± 5.4 | 1000.0 ± 24.2 | 27.5 ± 1.9 | 2000.0 ± 27.2 | 1700.0 ± 35.5 | 250.0 ± 13.5 | 2100.0 ± 93.0 | 1850.0 ± 71.3 | 381.0 ± 8.9 |
| **M1 NInv4** | 275.4 ± 4.5 | 1158.5 ± 25.7 | 27.0 ± 3.7 | 1700.0 ± 20.7 | 1588.3 ± 17.3 | 200.0 ± 15.7 | 1950.0 ± 29.1 | 1850.0 ± 30.1 | 350.0 ± 10.7 |
| **M1 NInv5** | 177.8 ± 7.78 | 1147.8 ± 15.8 | 27.2 ± 6.6 | 805.8 ± 17.6 | 1500.0 ± 62.6 | 200.0 ± 7.7 | 1100.0 ± 45.2 | 1700.0 ± 21.3 | 350.0 ± 6.1 |
| **M1 NInv6** | 230.0 ± 8.5 | 1200.0 ± 25.7 | 41.0 ± 4.3 | 1000.0 ± 41.1 | 1423.3 ± 60.4 | 82.0 ± 10.8 | 1555.5 ± 30.6 | 1582.9 ± 46.5 | 115.9 ± 13.7 |
|  |  |  |  |  |  |  |  |  |  |
| **M28 Inv1** | 96.6 ± 3.9 | 980.5 ± 21.1 | 70.0 ± 6.5 | 656.3 ± 17.6 | 1423.2 ± 43.4 | 83.4 ± 4.3 | 1196.8 ± 52.7 | 1517.9 ± 20.9 | 105.9 ± 13.7 |
| **M28 Inv2** | 57.7 ± 1.26 | 1134.7 ± 16.9 | 68.3 ± 5.1 | 364.5 ± 18.1 | 1408.4 ± 18.6 | 80.9 ± 2.5 | 1102.8 ± 78.4 | 1516.2 ± 43.7 | 150.0 ± 4.5 |
| **M28 Inv3** | 258.1 ± 6.1 | 501.9 ± 15.8 | 48.4 ± 4.5 | 617.5 ± 22.8 | 600.9 ± 10.3 | 95.6 ± 9.7 | 741.7 ± 11.0 | 628.7 ± 25.8 | 165.5 ± 8.7 |
| **M28 Inv4** | 371.7 ± 4.3 | 1049.6 ± 12.9 | 28.8 ± 1.8 | 714.4 ± 23.3 | 1212.4 ± 46.3 | 46.3 ± 3.2 | 1113.9 ± 94.2 | 1316.7 ± 23.8 | 101.8 ± 6.7 |
| **M28 Inv5** | 300.0 ± 11.1 | 829.8 ± 30.2 | 27.3 ± 3.2 | 764.1 ± 34.0 | 1282.8 ± 58.4 | 36.4 ± 1.7 | 1111.1 ± 39.8 | 1353.7 ± 21.5 | 104.3 ± 3.9 |
| **M28 Inv6** | 89.5 ± 1.1 | 248.1 ± 12.7 | 20.8 ± 2.5 | 167.1 ± 2.7 | 337.0 ± 16.2 | 22.6 ± 3.6 | 410.0 ± 11.9 | 616.8 ± 11.9 | 28.5 ± 1.5 |
| *Table S3 continued* | | | | | | | | | |
| **M28 Inv7** | 72.4 ± 7.2 | 1006.7 ± 16.5 | 11.0 ± 1.5 | 289.8 ± 24.7 | 1507.9 ± 53.6 | 30.0 ± 2.3 | 1116.3 ± 76.9 | 1613.6 ± 63.5 | 185.0 ± 2.2 |
| **M28 Inv8** | 126.3 ± 2.1 | 1413.2 ± 10.9 | 23.5 ± 1.7 | 486.5 ± 17.9 | 1532.3 ± 15.1 | 64.0 ± 2.9 | 1158.0 ± 50.7 | 1616.7 ± 20.3 | 343.4 ± 18.5 |
| **M28 NInv1** | 200.3 ± 7.4 | 976.1 ± 21.1 | 11.9 ± 1.6 | 249.4 ± 17.8 | 1070.5 ± 88.8 | 30.4 ± 2.5 | 976.8 ± 61.3 | 1215.8 ± 41.1 | 185.1 ± 20.3 |
| **M28 NInv2** | 234.7 ± 4.6 | 1006.7 ± 24.0 | 15.4 ± 2.8 | 802.4 ± 18.5 | 1300.6 ± 71.1 | 92.2 ± 5.2 | 1035.9 ± 36.9 | 1408.9 ± 38.6 | 238.6 ± 16.4 |
| **M28 NInv3** | 252.9 ± 3.8 | 802.8 ± 12.6 | 18.8 ± 3.2 | 543.4 ± 16.6 | 896.4 ± 16.2 | 96.3 ± 5.5 | 1001.9 ± 23.9 | 1000.0 ± 27.1 | 172.4 ± 12.4 |
| **M28 NInv4** | 336.7 ± 12.7 | 858.5 ± 27.7 | 29.6 ± 3.1 | 655.2 ± 27.1 | 1241.0 ± 54.3 | 36.8 ± 2.8 | 1250.6 ± 23.7 | 1430.4 ± 93.5 | 55.7 ± 4.3 |
| **M28 NInv5** | 165.0 ± 8.6 | 685.4 ± 13.5 | 28.2 ± 2.5 | 553.7 ± 21.3 | 1089.0 ± 35.6 | 32.9 ± 3.3 | 1049.7 ± 50.7 | 1301.4 ± 56.3 | 56.5 ± 4.9 |
|  |  |  |  |  |  |  |  |  |  |
| **M89 Inv1** | 200.9 ± 5.7 | 865.8 ± 10.7 | 119.7 ± 7.5 | 484.4 ± 43.1 | 1278.6 ± 28.9 | 67.5 ± 2.5 | 1000.0 ± 41.5 | 1580.9 ± 14.1 | 62.4 ± 2.1 |
| **M89 Inv2** | 140.8 ± 11.8 | 748.1 ± 10.3 | 71.2 ± 5.1 | 458.7 ± 36.1 | 1201.4 ± 24.4 | 46.4 ± 6.2 | 1264.2 ± 39.5 | 1505.1 ± 40.9 | 47.5 ± 16.1 |
| **M89 Inv3** | 191.3 ± 9.0 | 768.2 ± 20.1 | 422.9 ± 15.1 | 472.0 ± 16.6 | 1268.2 ± 23.1 | 51.9 ± 7.3 | 1566.1 ± 45.0 | 1569.3 ± 52.9 | 51.7 ± 9.5 |
| **M89 Inv4** | 215.1 ± 11.5 | 865.8 ± 25.5 | 85.7 ± 4.4 | 863.5 ± 36.7 | 1312.2 ± 53.2 | 100.0 ± 14.3 | 1453.1 ± 96.8 | 1546.7 ± 89.1 | 250.0 ± 9.5 |
| **M89 Inv5** | 191.2 ± 7.3 | 742.5 ± 28.4 | 48.5 ± 6.1 | 702.5 ± 19.8 | 1297.7 ± 35.7 | 54.7 ± 11.6 | 1819.6 ± 97.6 | 1544.4 ± 76.1 | 61.9 ± 7.3 |
| **M89 Inv6** | 128.7 ± 10.6 | 700.9 ± 19.9 | 71.6 ± 8.6 | 651.1 ± 40.7 | 1243.6 ± 50.7 | 55.0 ± 6.2 | 1313.3 ± 80.5 | 1489.4 ± 64.6 | 62.9 ± 8.2 |
| **M89 NInv1** | 94.6 ± 2.5 | 689.6 ± 19.4 | 57.5 ± 7.3 | 388.2 ± 14.8 | 1128.8 ± 38.3 | 52.2 ± 6.4 | 1324.1 ± 37.1 | 1534.0 ± 72.8 | 54.5 ± 7.1 |
| **M89 NInv2** | 443.5 ± 2.4 | 615.8 ± 15.8 | 193.6 ± 5.9 | 667.6 ± 67.5 | 1113.2 ± 35.8 | 54.4 ± 5.9 | 1075.7 ± 91.2 | 1412.0 ± 97.5 | 53.2 ± 5.7 |
| **M89 NInv3** | 88.8 ± 3.2 | 538.5 ± 17.4 | 104.1 ± 8.5 | 483.4 ± 24.8 | 874.3 ± 21.9 | 69.6 ± 7.8 | 901.9 ± 85.1 | 1196.6 ± 47.6 | 58.9 ± 2.9 |
| **M89 NInv4** | 52.0 ± 3.3 | 569.5 ± 9.4 | 69.9 ± 6.7 | 566.7 ± 19.7 | 1143.6 ± 47.4 | 53.9 ± 5.7 | 972.7 ± 20.5 | 1321.3 ± 74.4 | 52.0 ± 3.7 |
| **M89 NInv5** | 43.9 ± 5.1 | 346.9 ± 17.7 | 172.3 ± 8.5 | 150.2 ± 6.6 | 724.9 ± 45.5 | 44.7 ± 9.3 | 729.6 ± 30.7 | 1053.0 ± 27.8 | 49.7 ± 1.5 |
| **M89 NInv6** | 110.8 ± 11.3 | 826.0 ± 15.8 | 131.2 ± 16.4 | 517.5 ± 18.0 | 1191.1 ± 48.4 | 61.3 ± 9.5 | 1130.4 ± 50.5 | 1264.5 ± 34.3 | 54.2 ± 8.5 |

The values indicated are the means ± SD of three independent experiments
